# Diet flexibility and growth of the early herbivorous juvenile crown-of-thorns sea star, implications for its boom-bust population dynamics

**DOI:** 10.1371/journal.pone.0236142

**Published:** 2020-07-20

**Authors:** Dione J. Deaker, Benjamin Mos, Huang-An Lin, Corinne Lawson, Claire Budden, Symon A. Dworjanyn, Maria Byrne

**Affiliations:** 1 School of Medical Sciences, The University of Sydney, Sydney, New South Wales, Australia; 2 National Marine Science Centre, School of Environment, Science and Engineering, Southern Cross University, Coffs Harbour, New South Wales, Australia; 3 School of Life and Environmental Sciences, The University of Sydney, Sydney, New South Wales, Australia; The University of Hong Kong, HONG KONG

## Abstract

The ecology of the early herbivorous juvenile stage of the crown-of-thorns sea star (COTS, *Acanthaster* spp.) is poorly understood, yet the success of this life stage is key to generating population outbreaks that devastate coral reefs. Crustose coralline algae (CCA) has been considered to be the main diet of herbivorous juveniles. In this study, we show that COTS can avail of a range of algal food. Juveniles were reared on CCA, *Amphiroa* sp., and biofilm, and survived for 10 months on all three diets. The juveniles fed CCA and *Amphiroa* sp. reached 15–16.5 mm diameter at ~ 6 months and maintained this size for the rest the experiment (an additional ~4 months). Juveniles fed biofilm grew more slowly and to a smaller maximum size (~3 mm diameter). However, when juveniles were switched from biofilm to CCA they resumed growth to a new asymptotic size (~13.5 mm, 13–20 months). In diet choice experiments, juveniles did not show a preference between *Amphiroa* sp. and CCA, but generally avoided biofilm. Our results show that juvenile COTS grew equally well on CCA and *Amphiroa* sp. and can subsist on biofilm for months. Some juveniles, mostly from the biofilm diet treatment, decreased in size for a time and this was followed by recovery. Flexibility in diet, growth, and prolonged maintenance of asymptotic size indicates capacity for growth plasticity in herbivorous juvenile COTS. There is potential for juvenile COTS to persist for longer than anticipated and increase in number as they wait for the opportunity to avail of coral prey. These findings complicate our ability to predict recruitment to the corallivorous stage and population outbreaks following larval settlement and the ability to understand the age structure of COTS populations.

## Introduction

The feeding ecology of predatory sea stars has long been recognised to have fundamental effects on community structure [1, 2]. In coral reef ecosystems, population outbreaks of the coral predator *Acanthaster* spp. (crown-of-thorns sea stars, COTS) are one of the leading drivers of coral loss [3–5]. Outbreaks of COTS are likely to be driven by the high success rates of their early life history stages, a characteristic of echinoderms that exhibit boom/bust population fluctuations [6]. Despite the notoriety of the adult starfish and their propensity for coral prey, juvenile COTS are initially herbivores. They remain cryptic in the reef infrastructure and rubble where they are thought to mainly feed on crustose coralline algae (CCA) before transitioning to a coral diet [7, 8]. Although the success of the early juvenile stage is key to generate outbreaks, the biology and ecology of this stage is poorly understood [9].

It is becoming increasingly apparent that there are multiple factors and inherent species traits that enable COTS to succeed on undisturbed reefs and to capitalise on anthropogenic disturbances [9, 10]. The terrestrial run-off or enhanced larval survival hypothesis has achieved the greatest traction in explaining outbreaks, and posits that larval success and recruitment are enhanced by eutrophy-driven increases in their phytoplankton food [11–13]. The predator-removal hypothesis proposes that overfishing has released COTS from top-down control [14]. This is supported by findings that a suite of target (fished) and non-target species prey on COTS gametes, larvae, juveniles, and adults [15–17], and that marine protected areas which would be expected to have a more intact fish guild are less prone to outbreaks [18–20]. Finally, as periodic outbreaks appear to be an inherent feature of COTS, it has been hypothesised that these are also a natural phenomenon [21] and, as the larvae evolved in oligotrophic tropical waters, the larval-resilience hypothesis posits that they are naturally resilient to food limitation [22, 23]. These hypotheses, especially the terrestrial run-off hypothesis, have formed a framework to inform management actions to mitigate COTS outbreaks.

Aside from the predator-removal hypothesis, the potential role of the early juvenile stage in the success of COTS populations has not been incorporated into hypotheses frameworks and is only beginning to be considered. Regardless of larval settlement, the survival of the juvenile stage is required to seed outbreaks [24]. For instance, nutrient run-off stimulates the growth of macroalgae that can overgrow coralline algae, the food for juveniles, and disruptions to benthic assemblages may impact their predator guild [25, 26]. In a recent study, it was found that herbivorous juvenile COTS are extremely resilient to coral scarcity and may accumulate for years as they wait for coral prey before seeding an outbreak [27].

For outbreaks to arise, algae-eating COTS juveniles must transition into coral predators. The ontogenetic switch from herbivory to carnivory in predatory sea stars requires morphological and physiological changes to achieve competence to avail of an animal diet [28]. Juvenile COTS have been shown to consume CCA for 4 6 months post settlement in laboratory studies [7, 29] and for 13–15 months in nature [30]. Similar to COTS, juveniles of the sympatric corallivore *Culcita novaeguineae* initially consume CCA and biofilms [31] and the temperate species *Stichaster australis* switches from CCA to bivalve prey at 15–28 months of age [32]. To become a competent corallivore, juvenile COTS need to achieve a minimum size of ~ 8 mm diameter, be able to digest the complex wax esters in coral tissue, and withstand stings from coral polyps [7, 30, 33].

At present, CCA is considered to be the main settlement substrate for COTS larvae and the food for early juveniles [34]. However, COTS are able to settle in the absence of CCA [22, 35], and are known to have a flexible diet in their larval [36, 37] and corallivorous [24, 38, 39] stages. Juveniles have been reported to eat biofilms [31, 40]. Biofilms are a ubiquitous food source in nature and are considered to be a cost-effective food source for juvenile sea stars [28]. In a review on starfish feeding ecology, 44 of 57 species (17/29 omnivorous species) initially feed on biofilms as juveniles [28].

Variation in the palatability, digestibility, nutritional content, and energetic value of different food sources affect sea star growth rates [28, 41], suggesting that differences in diet may have important consequences for the growth of juvenile COTS. To determine if the early benthic stage of COTS has dietary flexibility, we conducted a long-term feeding experiment where cohorts of juveniles were reared on CCA, biofilm, and a second tropical calcifying coralline alga, *Amphiroa* sp., that has a geniculate form. Over 1–2 years, the growth, maximum size, and arm number were quantified. As biofilms are eaten by many juvenile sea star species and are ubiquitous in nature [28, 42], we determined if COTS can be sustained on a biofilm diet until more optimal food (e.g. CCA) becomes available. Juveniles that had been raised on biofilm were switched to CCA to determine if their growth would recover to match that of the juveniles initially provided with coralline algae and if they could reach the size threshold required to transition to a coral diet. To determine if diet history affects diet choice and test whether CCA is the preferred diet of juvenile COTS, we offered the juveniles from the different food treatments a choice of all three diets.

Juvenile sea stars are well known to have variable growth post-settlement with cohorts comprising of fast and slow growing individuals as well as prolonged growth stasis [43, 44]. We expected that juvenile COTS would grow well on CCA as shown in previous studies [7, 8], but that they would also exhibit potential as flexible opportunistic feeders and consume other food sources. We also expected that juveniles would exhibit different growth rates and maximum sizes on the diets provided and that this variability in size would be magnified across the different treatments. We determined the capacity for growth stasis and long-term size persistence of juvenile COTS in the absence of coral prey, a key consideration with respect to the sources of outbreaks and in interpretation of the age structure of COTS populations.

## Methods

*Acanthaster* comprises a species complex found throughout the tropical Indo-Pacific with uncertain taxonomy [45, 46]. We refer to this species as COTS or *Acanthaster* sp..

Adult *Acanthaster* sp. were obtained from the Australian Marine Tourism Operator Association who have permission from the Great Barrier Reef Marine Park Authority to harvest crown-of-thorns. They were collected near Cairns, North Queensland, Australia (16°550’S, 145°460’E) and transported to the National Marine Science Centre (NMSC), Southern Cross University in Coffs Harbour, NSW, where they were maintained in flow-through aquaria at 26–27°C. Two males and two females were spawned. Gonads were removed through small incisions in the body wall. Ovaries were placed in the ovulatory hormone 10^−4^ *M* 1-methyl-adenine in 1 μm UV-filtered sea water (FSW) for 30–45 min until the eggs had matured, confirmed by checking for germinal vesicle breakdown. Sperm was collected by macerating the testes and was activated in seawater. Eggs and sperm were checked microscopically for quality. Equal amounts of the gametes were pooled between the males and the females and with an egg to sperm ratio of ~1:100 were fertilised ensuring at least 95% fertilisation success as checked microscopically for the fertilization envelope around the eggs. These cultures were established in late February.

The larvae were reared in two 300-L cylindro-conical tanks at 26°C [47] in FSW that was replaced every 1–2 days. They were fed daily with 25–40 ×10^3^ cells ml^-1^ of the tropical microalga *Proteomonas sulcata* once the gut was formed (~48 h post fertilisation). When the larvae reached the brachiolaria stage (16–18 days old), polycarbonate sheets covered in CCA were placed into the tanks to induce settlement. Over the next 21 days settlement was asynchronous. Juveniles 1–2 mm diameter (≤ 3 months old) were collected from the sheets for the start of the experiment.

### Juvenile feeding experiment

**Fig 1:**
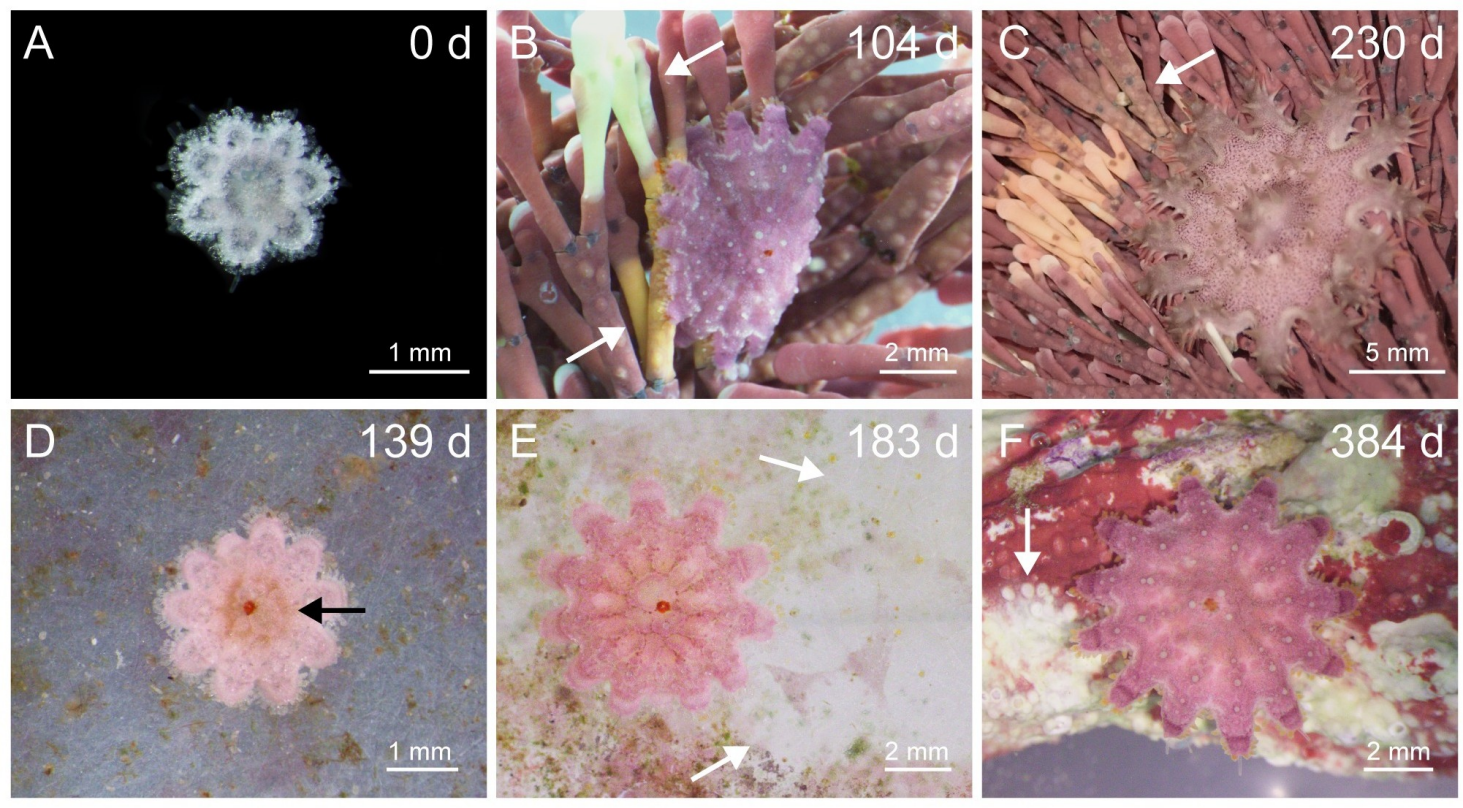
Herbivorous juvenile crown-of-thorns sea stars, *Acanthaster* sp., feeding on coralline algae and biofilms. (A) Juveniles at the start of the feeding experiment. (B-C) Juveniles raised on *Amphiroa* sp.. The juvenile in (B) is wrapped around the fronds to feed. (D-E) Juveniles raised on biofilm for 292 d, and then provided with crustose coralline algae (CCA) (F, 92 d on CCA). The number in the top right corner of each panel represents the number of days since the experiment commenced. The black arrow (D) indicates a green stomach associated with eating biofilm. The white arrows indicate feeding scars on *Amphiroa* sp. (B-C), the biofilm plate (E), and CCA (F).

The juveniles were randomly distributed into plastic pots (4 cm Ø) and were fed crustose coralline algae (CCA), *Amphiroa* sp., or biofilm for 10 months (292 d) (n = 20 per diet). There was no significant difference in the initial size of the juveniles between the food treatments (mean ± SE = 1.67 ± 0.04 mm Ø, n = 30, F_2, 57_ = 0.42, p = 0.66, Fig 1A). The CCA was cultured on polycarbonate sheets and small pebbles at ~26°C. Biofilm was grown on plastic sheets in tanks at NMSC for > 2 y, and contained a mix of naturally occurring bacteria, diatoms, algae, and multi-cellular animals. The polycarbonate sheets were cut into 2×3 cm pieces. *Amphiroa* sp. was collected at low tide from Charlesworth Bay, Coffs Harbour (30° 16’ 7” S, 153° 8’ 13” E), and rinsed in freshwater to remove motile invertebrates. After 292 d, the juveniles fed biofilm were switched to CCA for an additional 304 d.

Pots were haphazardly distributed in a flow-through system that delivered 1 μm-UV FSW through an individual dripper into each pot. The drippers were adjusted daily to maintain flow and 26°C (mean = 26.01°C, SD = 0.30°C, n = 228) with temperature monitored using a Hach^®^ HQ40d multi-controller with a Hach^®^ PHC101 probe. Algal food was replaced frequently to ensure that the juveniles were fed *ad libitum*. When the juveniles reached 8 mm Ø they were transferred into larger plastic pots (6 × 4 × 2 cm) to supply sufficient food. The pots were washed and replaced every two weeks to prevent fouling. The juveniles were monitored daily for survival and condition. On day 143, the position of the juveniles in their pots (on food vs. not on food) was recorded once a day for 9 d as a proxy for time spent feeding.

To follow growth and development, juveniles were photographed every 2–4 weeks using an Olympus DP25 digital camera mounted on an Olympus SZX7 dissecting microscope. When the juveniles exceeded 10 mm Ø, they were photographed using an Olympus tough TG-5 mounted on a GorillaPod (Joby) stand. The number of arms were counted, and the diameter was measured from photographs using ImageJ software (ver. 1.52a, NIH, USA). Growth rates were calculated across the time points where their diameter was increasing at a constant, linear rate. Growth data for juveniles fed CCA are from Deaker et al. [27].

### Diet choice experiment

Juveniles raised on CCA, *Amphiroa* sp., and biofilm were offered a choice of each of the three food sources provided simultaneously. The juveniles raised on *Amphiroa* sp. and CCA were offered approximately 4 cm^2^ of CCA, *Amphiroa* sp., and biofilm concurrently in individual pots (6 × 4 × 2 cm). As the biofilm juveniles were smaller, they were offered 1 cm^2^ of each substrate in individual pots (~4 cm Ø). Choice experiments were performed first for fed juveniles (n = 10, 260 d). One month later these juveniles were starved for three days and the experiment was performed again (n = 10, 292 d). Each juvenile was placed in the centre of a pot approximately equal distances from the different substrates. The juvenile’s initial choice (time = 0) was recorded and their movement was then tracked by recording their position hourly over 48 h without disturbing the pots. As it was not possible to measure the feeding scars on the CCA covered pebbles or distinguish feeding scars on the biofilm and *Amphiroa* sp. to measure feeding rates, the amount of time the juveniles spent on a particular substrate was used as the response metric. The pots housing the juveniles remained in the previously described flow-through seawater system throughout the choice experiments.

### Statistical analysis

Analyses of juvenile growth, survival and arm number data were performed in R (version 3.4.3) [48]. The initial size of juveniles was analysed using a one-way ANOVA (lm function, statistics package). The data was homoscedastic (Levene’s test, p > 0.05) and normally distributed which was confirmed by visually inspecting the distribution of residuals on a q-q plot. Survival data were analysed using a log-rank test and specific differences between groups were tested using post-hoc pairwise comparisons (packages: survival and survminer).

Arm number and diameter were compared among juveniles fed CCA, *Amphiroa* sp., and biofilm for 292 d, and between juveniles fed CCA or *Amphiroa* sp. for 292 d and biofilm-CCA at 586 d to see if arm number and size of the biofilm cohort recovered. These data were not normally distributed and were analysed using Kruskal–Wallis test by ranks. For the biofilm cohort, arm counts over time were analysed to determine if the number increased during the biofilm and CCA phases. These data were also not normally distributed and were analysed using a repeated measures one-way ANOVA (lme function, nlme package [49]) with a rank transformation as the same juveniles were measured over time. Post-hoc analysis were computed for significant main effects using Tukey-adjusted pairwise comparisons (Kruskal tests: PMCMR package [50]; one-way ANOVA: emmeans package [51]. The coefficient of variation, ($CV=100\times \frac{standard\;deviation}{mean}$), was calculated to determine the variability of the diameter of juveniles within a diet treatment and across all diets after 292 days. All graphs were made using ggplot 2 [52].

To determine if COTS showed a preference for particular algal substrates due to diet history, the amount of time each juvenile spent on *Amphiroa* sp., biofilm, CCA, or no choice (bare substrate) in both the fed and starved experiments were ranked and the ranks were analysed by Friedman’s rank test using the IBM SPSS Statistics program (v. 25.0). Replicates from each diet treatment (*Amphiroa* sp., CCA, biofilm) and food availability treatment (fed, starved) were examined separately. Post-hoc Wilcoxon tests were used where Friedman’s test indicated significant differences among substrates (p < 0.05).

## Results

### Juvenile feeding and growth

White and orange/yellow feeding scars were present on CCA and *Amphiroa* sp. (Fig 1B, 1C and 1F). The juveniles on *Amphiroa* sp. wrapped themselves around the fronds as they fed (Fig 1B). Feeding scars were rarely identified on biofilm plates, although the juveniles had green-brown stomachs indicating that they were feeding (Fig 1D and 1E). Over a nine-day period (from day 143), those in the CCA and the *Amphiroa* sp. treatments were positioned on their food for 85.2% (SE, ± 3.2%, n = 19) and 98.1% (± 1.3%, n = 18) of the time, respectively. The biofilm juveniles were only recorded on their food for 5.6% of the time (± 1.9%, n = 18). Juveniles exhibited a fleeing response when disturbed in their pots climbing up to the water surface and then float at the surface oral-side up supported by the water tension and with their tube feet extended. If the tension was disturbed such as by a water drop the juvenile detached and fell to the bottom of the container. This behaviour was observed ~ 40 times in juveniles 1.5–18 mm diameter.

**Fig 2:**
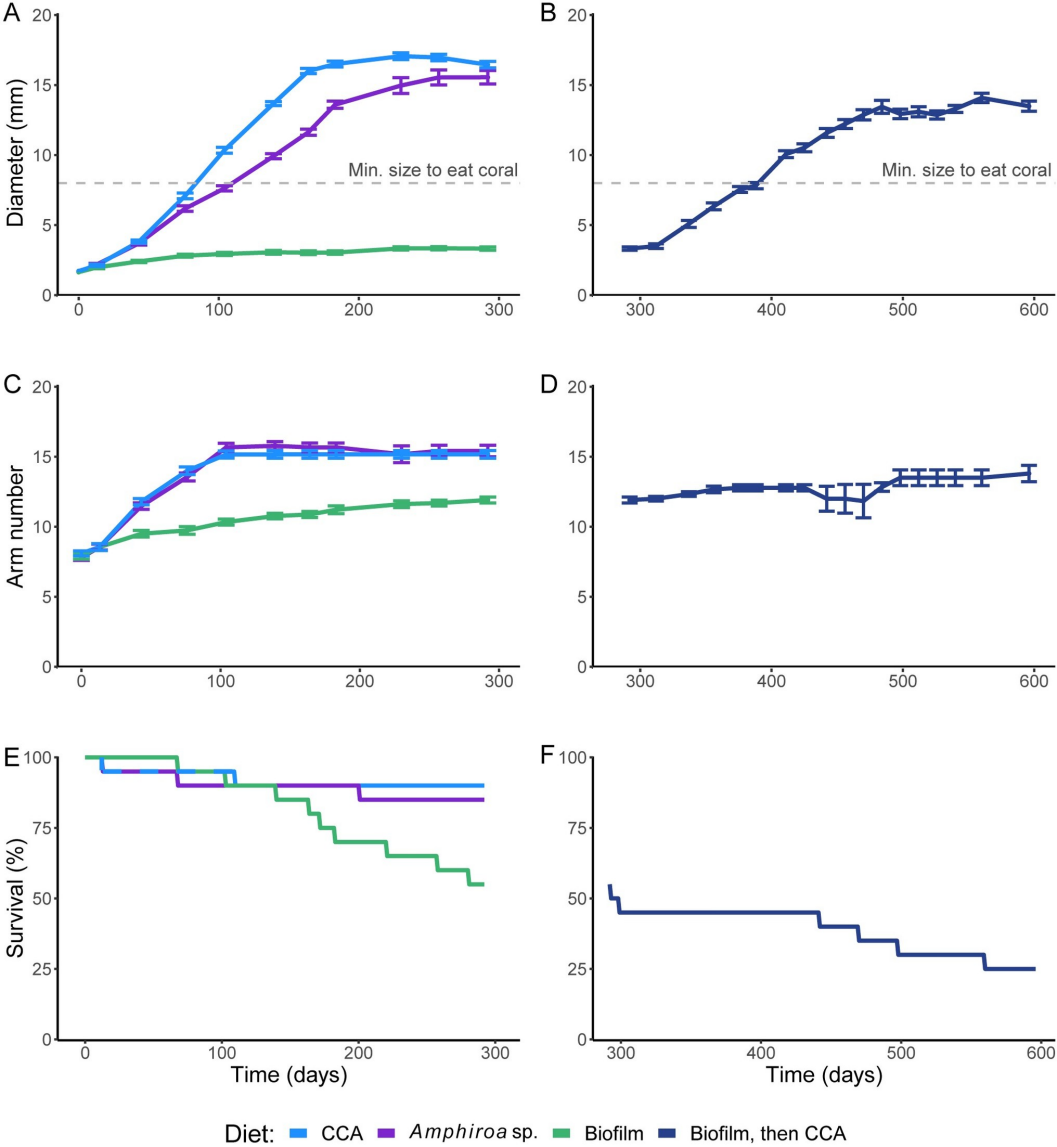
The growth and survival of herbivorous juvenile crown-of-thorns sea stars on three different diets. (A,B) The diameter (mean ± SE), (C-D) number of arms (mean ± SE), and (E-F) survival (%) of juveniles fed crustose coralline algae (CCA), *Amphiroa* sp., or biofilm for 292 d (left panels) and juveniles that were raised on biofilm and then fed CCA for an additional 304 d (right panels). The horizontal dashed lines (A-B) represent the approximate minimum size that juveniles can transition to a coral diet [7]. Juveniles were ≤3 months old at the start of the experiment (T_o_).

The growth of juveniles on a diet of CCA was initially exponential until they reached an inflexion point at ~ 10 mm, plateauing after 164 d (mean Ø ± SE, 16.00 ± 0.19 mm, Fig 2A). The growth of juveniles on a diet of *Amphiroa* sp. was linear until the growth curve flattened after 183 d (13.59 ± 0.26 mm, Fig 2A). Initial growth rates were similar on the CCA and *Amphiroa* sp. diets, 0.05 mm/day (0–43 d) and 0.06 mm/day (0–183 d), respectively. After 43 d, the growth rate of the CCA cohort increased to 0.10 mm/day (43–164 d). Growth of the biofilm-fed cohort was slow (0.01 mm/day, 0–76 d) with the maximum size plateaued after 76 d (2.81 ± 0.12 mm Ø, Fig 2A). After biofilm-fed juveniles were switched to CCA they resumed growth and increased in size (0.06 mm/day, 20–187 d on CCA) and this plateaued at 470 d (12.87 ± 0.36 mm Ø, Fig 2B).

There was no difference in the final diameter of the cohorts fed CCA (16.45 ± 0.23 mm) and *Amphiroa* sp. (15.55 ± 0.48 mm), while those fed biofilm were smaller (3.32 ± 0.12 mm, χ^2^ = 26.00, df = 2, p < 0.0001). Arm number followed a similar pattern as diameter, with the maximum arm number reached after 139 d on a diet of CCA (15.17 ± 0.27 arms, n = 18, Fig 2C) and *Amphiroa* sp. (15.41 ± 0.41 arms, n = 17, Fig 2C). The juveniles fed coralline algae had significantly more arms than the biofilm cohort (11.91 ± 0.21 arms, χ^2^ = 22.27, df = 2, p < 0.0001, Fig 2C). By 292 d, the coefficient of variation of size in the cohort of juveniles fed CCA, *Amphiroa sp*. and biofilm was 6.0%, 12.8% and 11.9% respectively, and 43.5% across all cohorts.

After switching from biofilm to CCA, the biofilm cohort remained significantly smaller than the juveniles from the other diet treatments (596 d, 13.50 ± 0.36 mm Ø, n = 5, χ^2^ = 13.61, df = 2, p = 0.001, post-hoc: CCA = *Amphiroa* sp. > biofilm-CCA). The increase in the number of arms of the juveniles fed biofilm was significant over time (F_27,270_ = 22.341, p < 0.001, post-hoc: 0 d < 292 d) and they developed more arms through the CCA phase reaching their maximum arm number after 498 d (13.50 ± 0.56 arms, n = 6, Fig 2D). Despite this, arm number during the CCA phase did not differ from the end of the biofilm phase (post-hoc: 292 d = 312–596 d), and at 596 d, biofilm-CCA juveniles had fewer arms than juveniles from the coralline algae treatments at 292 d (χ^2^ = 4.62, df = 1, p = 0.031).

### Survival and observations of body damage and recovery

The survival of juveniles was significantly different between treatments (χ^2^ = 7.3, df = 2, p = 0.003). However, the reduced survival of the biofilm treatment was not significant (p > 0.05), likely due to the limited power of the post-hoc pairwise comparison (low sample sizes within treatments). Survival of the juveniles fed CCA and *Amphiroa* sp. was high (90% and 85%, respectively, Fig 2E). One juvenile fed *Amphiroa* sp. (16 arms, 9.28 mm Ø, 139 d) lost arms with the number of arms reduced to 14 by 214 d (13.75 mm Ø) and 7 by 230 d (7.26 mm Ø). This juvenile recovered to have 11 arms by 257 d (8.44 mm Ø).

In contrast to the coralline algae diets, only 55% of the biofilm fed juveniles survived to day 292. When the surviving juveniles (n = 11) were switched to CCA, another 6 juveniles died by 596 d (Fig 2F). The 11 juveniles fed biofilm that survived variably regressed and recovered in size through the experiment. The average decrease in their diameter was 11.07% (SE, ± 3.5%, range: 0.14–65.47%). One of the biofilm fed juveniles that was switched to CCA lost half of its body and 6 arms by bisection of the central disk (442 d, 12 arms and 11.69 mm to 10.06 mm Ø and 6 arms) and this was followed by regeneration over the next 56 days to form 10 new arms (16 arms, 11.66 mm Ø, 498 d).

### Diet choice experiment

**Fig 3:**
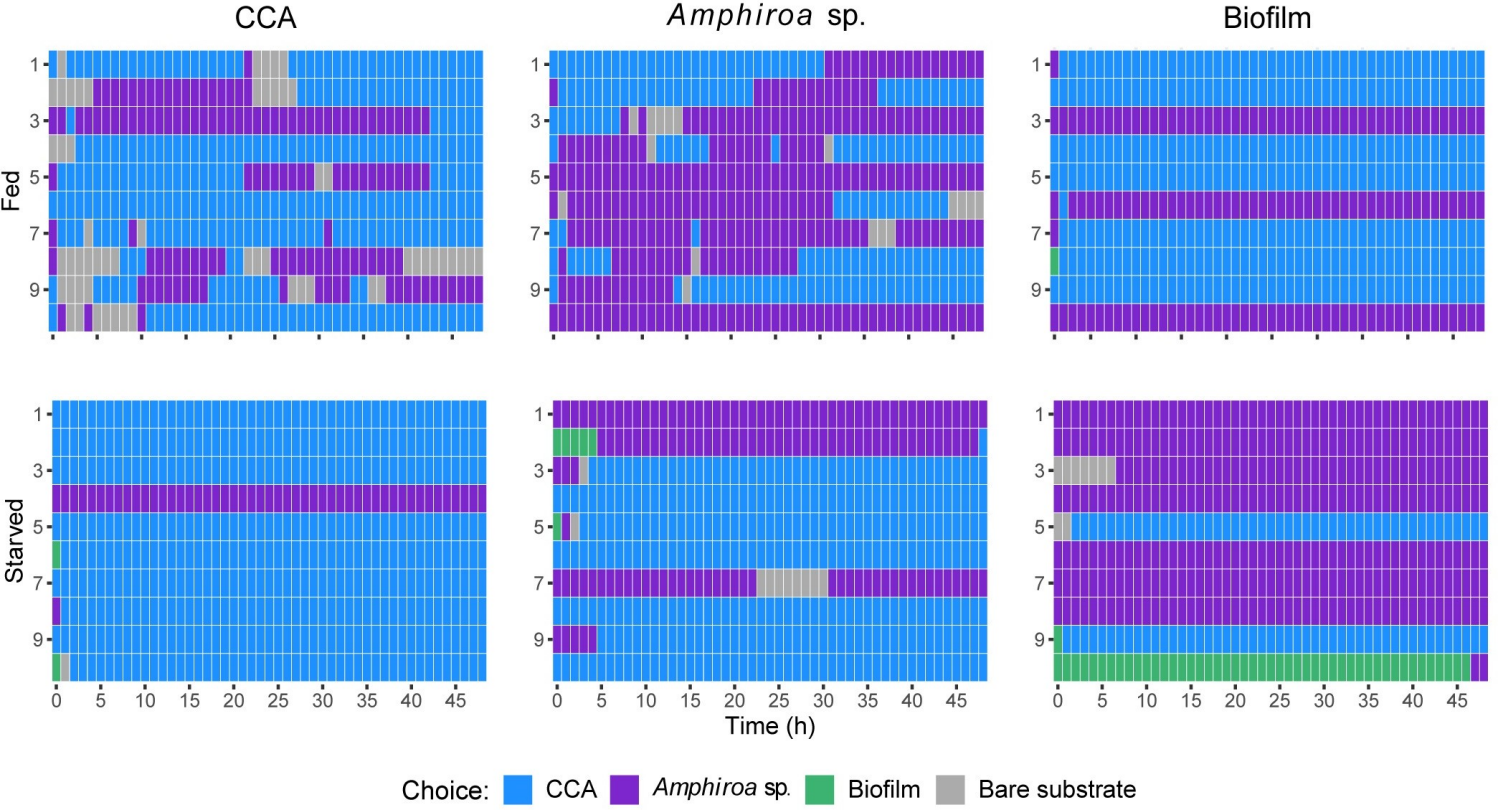
The effect of diet and starvation on the diet choice of juvenile crown-of-thorns sea stars. Juveniles raised on a diet of crustose coralline algae (CCA), *Amphiroa* sp., or biofilm for 292 d were offered three food sources simultaneously before (Fed, 260 d) and after they were starved for 3 d (Starved, 292 d). Their position on the algal foods or bare substrate were recorded each hour for 48 h. Each row represents the position of one individual juvenile over time (n = 10 per treatment), and each box represents the location of that juvenile recorded every hour.

Regardless of juvenile diet, CCA and *Amphiroa* sp. was preferred over biofilm and bare substrate (Fig 3). In the fed experiment, the amount of time juveniles spent on CCA and *Amphiroa* sp. did not differ (Table 1). Starvation for 3 d impacted the choice of the juveniles raised on CCA that spent more time on CCA and the juveniles fed biofilm that spent more time on *Amphiroa* sp. (Fig 3, Table 1). Juveniles were rarely recorded on biofilm and were observed to walk over and off this substrate.

**Table 1:** The mean time (%) a juvenile spent on different algal foods over a 48 h period (n = 10 per treatment).

Diet	Experiment		Choice (%)				Friedman’s tests		
			CCA	*Amphiroa* sp.	Biofilm	No choice	χ^2^ (df = 3)	p-value	Wilcoxon test post-hoc
CCA		*Fed*	61.04	27.50	0.00	11.46	19.36	< 0.001	CCA = *Amphiroa* sp. > *Amphiroa* sp. = bare substrate > biofilm
		*Starved*	89.79	10.00	0.00	0.21	21.67	< 0.001	CCA > *Amphiroa* sp. = biofilm = bare substrate
*Amphiroa* sp.		*Fed*	35.42	61.04	0.00	3.54	21.20	< 0.001	*Amphiroa* sp. = CCA > biofilm = bare substrate
		*Starved*	68.33	28.75	0.83	2.08	12.35	< 0.006	CCA = *Amphiroa* sp. > *Amphiroa* sp. = biofilm = bare substrate
Biofilm		*Fed*	70.21	29.79	0.00	0.00	15.38	< 0.002	CCA = *Amphiroa* sp. > *Amphiroa* sp. = biofilm = bare substrate
		*Starved*	19.79	69.17	9.58	1.46	10.65	< 0.014	*Amphiroa* sp. > CCA = biofilm = bare substrate

Preference experiments were carried out using juveniles raised on crustose coralline algae (CCA), *Amphiroa* sp., or biofilm for 292 d. Juveniles were simultaneously offered CCA, *Amphiroa* sp., and biofilm before (*Fed*, 260 d) and after they were starved for 3 d (*Starved*, 292 *d*), and their position on the algal foods or bare substrate recorded every hour for 48 h. The preference of juveniles for the algal foods provided, indicated by the amount of time spent on each substrate, was compared using Friedman’s tests.

Starvation affected the behaviour of juveniles reared on CCA and *Amphiroa* sp.. Fed juveniles were not always recorded on food and explored both CCA and *Amphiroa* sp., whereas starved juveniles from the same treatments typically remained on the substrate chosen initially or within the first few hours (Fig 3). Juveniles reared on biofilm tended to stay with their initial choice regardless of whether they had been starved (Fig 3).

## Discussion

The dietary flexibility and growth variability of juvenile COTS seen here provides new insights with respect to recruitment into the coral eating adult stage. Our results highlight the diet-dependent growth rates of the herbivorous juvenile stage of COTS, their opportunistic nature to avail of a range of algal food as well as their resilience to starvation by subsisting on biofilm. Although juvenile COTS are known for their consumption of CCA [30], they also occur on articulated coralline algae [53], a more complex habitat. Habitat complexity is strongly linked to survival of juvenile echinoderms [54, 55] and by wrapping around the fronds they may be harder to detect by both researchers and predators. Variable diet may impact the growth and habitat distribution of juvenile COTS complicating our ability to model the dynamics of COTS outbreaks.

### Diet, growth, and behaviour

**Fig 4:**
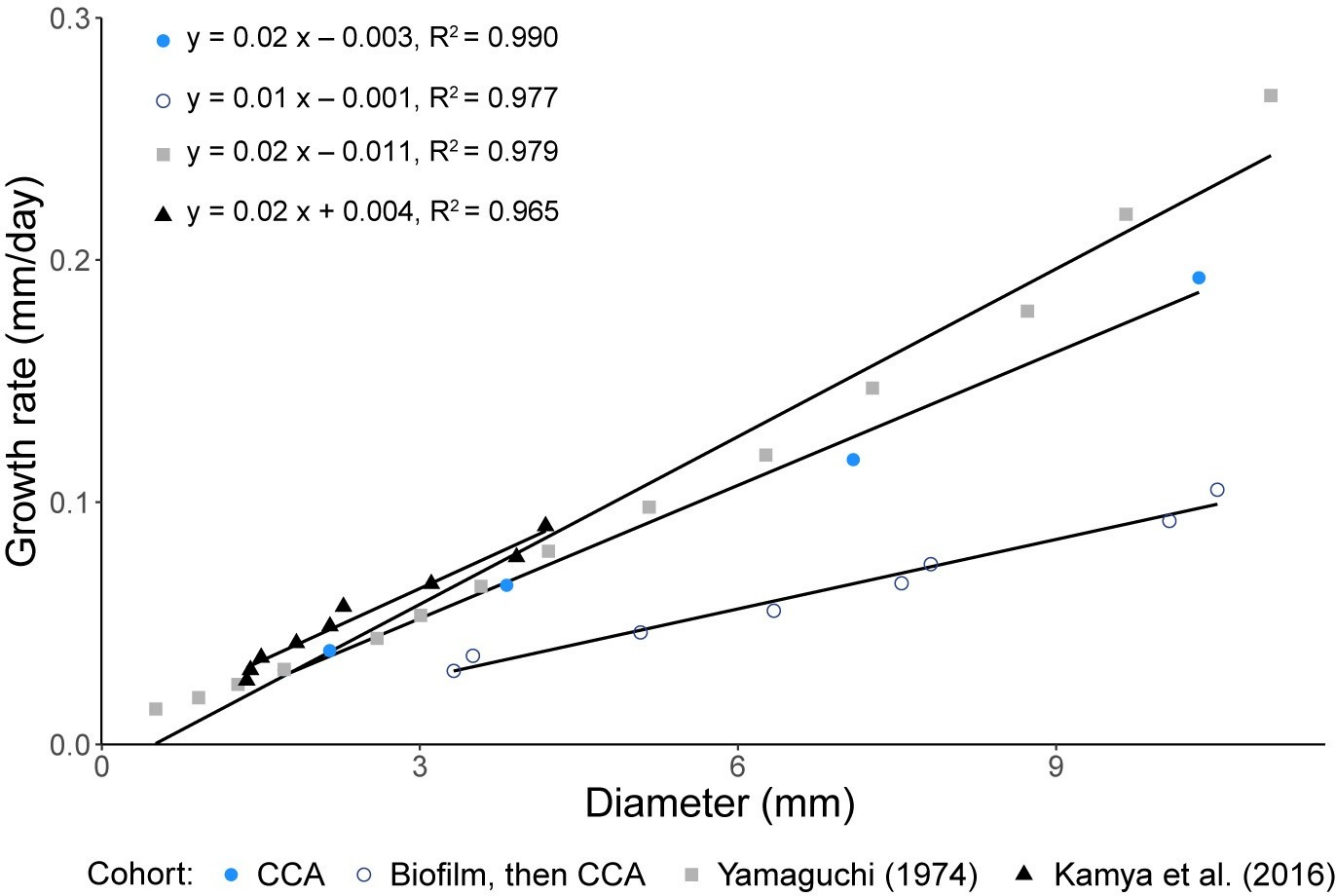
The mean growth rate of herbivorous juvenile crown-of-thorns sea stars during their exponential growth phase. Four cohorts of juveniles were raised on crustose coralline algae (CCA) in the laboratory. The data were obtained from Yamaguchi [7], Kamya et al. [58], and this study (diets: CCA and Biofilm, then CCA) (S1 Appendix). The growth rate with respect to juvenile diameter was determined by creating an exponential model of the change in mean diameter of each cohort (S1 Table). See supplementary material for equations and R^2^ values (S2 Table).

Geniculate coralline algae (*Amphiroa* sp.) was readily consumed and supported the growth of juveniles just as well as CCA. Despite a slower growth rate, juveniles fed *Amphiroa* sp. reached and maintained a maximum size of ~16 mm as with juveniles fed CCA here and in previous studies (18 mm [8], 10–18 mm [56]), a size that can be maintained for 6.5 years before transitioning to coral [27]. The faster, exponential growth rate of juveniles fed CCA in this study was similar to two previous studies (~0.02 mm day^-2^, Fig 4; S1 Appendix), although after being fed biofilm for 10 months, the growth rate on CCA was reduced (~0.01 mm day^-2^). If there is no change in diet, growth appears to be predictable. Otherwise, our results suggest that any changes in diet and diet history impacts growth and this may vary with the other types of coralline algae that they have been found associated with [53]. This may explain the variable size of juveniles in nature [57] confounding size-at-age models.

Biofilm was a comparatively poor diet for juvenile COTS, yet they were able to survive on it for at least 10 months. As the growth on biofilm was minimal, these juveniles were only able to reach the size threshold to be competent corallivores on provision with CCA, ~19 months later than those fed coralline algae. Biofilm appears to be sufficient to maintain physiological processes for COTS and is the primary diet of a number of starfish species at the juvenile stage [2, 28], although its nutritional quality may vary at small spatial scales in nature depending on the species of cyanobacteria present and with variable protein, carbohydrate and caloric levels [28, 59]. Mortality of the biofilm juveniles was high (45%) and some of them shrank, indicating they may have stopped feeding for periods of time. Decrease in size is a common response to food scarcity and environmental stress among echinoderms, including immature COTS [7, 41, 60]. The juveniles fed biofilm did not reach the size threshold to transition to a coral diet until after they had switched diets to consume CCA for ~4 months. Importantly, there was a negative carry over effect of eating biofilm that stunted their growth after switching to CCA. Biofilm-raised juveniles did not grow to the same size or have the same number of arms as juveniles that ate coralline algae from the outset. As smaller individuals are more vulnerable to predation, juveniles that feed primarily on biofilm and remain smaller for a longer periods of time are likely to suffer greater mortality [15].

An interesting observation was that one juvenile split in half through the central disk, reminiscent of fissiparity in other sea stars [61] and cloning in COTS larvae [62]. One half perished and the surviving half regenerated to a normal juvenile with 16 arms, four more than it had initially. Once they have stopped adding arms on an algal diet, arm number does not appear to change when they switch to a coral diet [27] and into adulthood [8] unless they undergo trauma as seen here. Adult COTS also show extensive abilities to regenerate allowing for the regrowth of body parts lost due to sublethal predation [63, 64]. Notably, predators were excluded from this system. Fissiparous echinoderms may discard part of their body (a “sacrificial half”) in response to a stressful environment or to reduce metabolic costs [65, 66] and so the response of this juvenile fed CCA after biofilm may have been stress related. Given this only occurred in one juvenile COTS, it is unclear whether this phenomenon has implications for population dynamics and COTS outbreaks. However, it does show the ability of juveniles to recover from trauma.

When disturbed, some juveniles climbed up the walls of their pots and floated oral side up supported by the water tension and detached from the surface when the tension was broken. Similar floating behaviour is reported for other juvenile sea stars in laboratory studies where it is suggested to be a dispersal mechanism for shallow water, intertidal species [67, 68]. It is unlikely that juvenile COTS would avail of such a dispersal mechanism as they are usually found in submerged habitats, although they do occur in the intertidal zone and shallow reef flats [69–71].

In the diet choice experiments, food choice was not related to diet history. Overall, juveniles reared on CCA, *Amphiroa* sp., and biofilm preferred the two coralline algae over biofilm. Juveniles fed consistently throughout the day and night although the light cycle was not controlled to mimic day/light experienced by juveniles in nature where they are suggested to be nocturnal feeders [30]. In the fed experiment, the smaller juveniles from the biofilm cohort typically remained with their initial choice, while the larger juveniles from the CCA and *Amphiroa* sp. cohorts explored the different substrates. Starved juveniles from all three cohorts prioritised feeding and remained with their initial choice. Smaller juveniles have limited mobility compared to larger individuals and tend to stay on their food to grow. This may also reduce predation risk [35, 40]. In a field study juvenile COTS were observed to migrate from algal to coral habitat at ~25 mm diameter [30]. It is likely that juveniles do not leave their food unless their food source becomes depleted or they are large enough and have enough energy to search for alternative food.

### Implications for growth dynamics and aging

The development of population and size-age relationship models are guided by the assumption that larval settlement and the post-larval diet of COTS is restricted to CCA [10, 69, 70]. The effects of different diets on growth complicates our ability to age juvenile COTS, Wilmes *et al*. [57] and Zann et al. [30] estimated the age of juveniles in field studies from a predicted month of settlement. In both studies, juvenile size became more variable over time and this was attributed to the timing of the transition from herbivory to corallivory, a transition that may be delayed for years [27, 56]. We now show that this may also be driven by a mixed algal diet. The pre-coral juveniles (20–32 mm Ø) in Zann et al. [30] were nearly 20–100% larger than the maximum sized reached by the juveniles fed coralline algae in the present study. The large size of these juveniles indicate that they may have been eating coral or availed of animal prey [8, 56]. Thus, the size-age relationship of juveniles in nature is difficult to discern.

Another prominent example of a boom/bust predatory sea star is *Asterias rubens* in the north Atlantic [6]. Population outbreaks of this sea star have been studied for decades because they have devastating effects on commercial bivalves [72]. Like COTS, outbreaks of *A*. *rubens* and other *Asterias* species are thought to be instigated by massive larval recruitment events [73]. For *Asterias*, when all available prey is consumed cannibalism leads to the demise of an outbreak, a phenomenon that is not reported for COTS. Similar to COTS, the growth of juvenile *A*. *rubens* varies depending on environmental conditions and the consistency of their food source [43, 60, 74]. In the absence of suitable prey, juveniles enter a ‘waiting stage’ for months and exhibit minimal growth until they have access to food [75]. Like COTS, juveniles of *A*. *rubens* and potentially other *Asterias* species may accumulate over time before seeding an outbreak.

The ability for growth stasis as well as the negative carryover effects due to diet suggests that growth of juvenile COTS in the field is likely to be indeterminate. This compromises our ability to age adults. As for the juveniles, the variable asymptotic sizes of adults on different reefs has been suggested to be due to the local environmental setting rather than their genotype [76]. Counting the seasonal pigment bands on COTS spines [77, 78] may only be useful to determine the time since maturity when COTS are suggested to start forming these bands [76]. Proposed age-growth relationships for COTS currently used for population modelling are likely to be only indicative. A prolonged study is needed to determine if there are carry over effects of the diet of herbivorous juvenile COTS for subsequent life stages and generations [8].

The variable duration of the herbivorous juvenile stage complicates our understanding of the bottom-up processes that drive population outbreaks. This is a key consideration for models linking outbreaks to terrestrial run-off events [5, 12, 79], as well as models of the population dynamics of COTS that have largely focused on the larval and corallivorous stages [12, 19, 80] with few considering the herbivorous juvenile [10, 81]. While all stages of the life cycle of COTS have to be successful to generate outbreaks, enhanced survival of the juvenile stage may be the rate determining factor governing recruitment into the adult population [24, 82].

Our results suggest that the feeding ecology of juvenile COTS exerts a major influence on the timing of population outbreaks. On a favourable diet, juvenile COTS exhibit rapid growth compared to sympatric sea star species due to their large stomach area [83], lower investment in a calcified body (e.g. *Linckia laevigata*, *Culcita noveaguineae*), and early ontogenetic diet shift to coral [84, 85]. These inherent traits of COTS biology may contribute to rapid population expansion. On the other hand, poor food conditions may be associated with slow population expansion of adults and the juveniles that can persist during food scarcity may accumulate in the reef infrastructure. This creates the possibility that reserve populations of juvenile COTS can delay their switch to corallivory as they wait for food conditions to improve, and thereby delay the appearance of an outbreak for years.

## Supporting information

S1 Appendix Model of the exponential growth phase of herbivorous juvenile crown-of-thorns sea stars (COTS) on a diet of crustose coralline algae (CCA). (DOCX)

S1 Table Exponential models fitted to the growth of four cohorts of juvenile crown-of-thorns sea stars. Four cohorts of juveniles were raised on a diet of CCA in this study (CCA and biofilm, then CCA), Yamaguchi [1], and Kamya et al. [2] where D = diameter (mm) and t = time (days). The equation was differentiated (D’) to determine the equation for the rate of growth. DF, degrees of freedom.
(DOCX)

S2 Table The linear relationship between the growth rate (mm/day) and the diameter juvenile crown-of-thorns sea stars. Four cohorts of juveniles were reared on CCA in this study (CCA, biofilm, then CCA), Yamaguchi [1], and Kamya et al. [2]. DF, degrees of freedom.
(DOCX)

S1 Data (XLSX)

## References

[1] Paine RT. Intertidal community structure experimental studies on the relationship between a dominant competitor and its principal predator. Oecologia. 1974;15(2):93–120. 10.1007/BF00345739 28308255

[2] Sloan NA. Aspects of the feeding biology of asteroids. Oceanogr Mar Biol. 1980;18:57–124.

[3] De'ath G, Fabricius KE, Sweatman H, Puotinen M. The 27-year decline of coral cover on the Great Barrier Reef and its causes. Proc Natl Acad Sci U S A. 2012;109(44):17995–9. 10.1073/pnas.1208909109 23027961 PMC3497744

[4] Rotjan RD, Lewis SM. Impact of coral predators on tropical reefs. Mar Ecol Prog Ser. 2008;367:73–91. 10.3354/meps07531

[5] Pratchett MS, Caballes CF, Rivera-Posada JA, Sweatman HPA. Limits to understanding and managing outbreaks of Crown-of-Thorns starfish (*Acanthaster* spp.). Oceanogr Mar Biol. 2014;52:133–200. 10.1201/b17143-4

[6] Uthicke S, Schaffelke B, Byrne M. A boom-bust phylum? Ecological and evolutionary consequences of density variations in echinoderms. Ecol Monogr. 2009;79(1):3–24. 10.1890/07-2136.1

[7] Yamaguchi M. Growth of juvenile *Acanthaster planci* (L.) in the laboratory. Pac Sci. 1974;28(2):123–38.

[8] Lucas JS. Growth, maturation and effects of diet in *Acanthaster planci* (L.) (Asteroidea) and hybrids reared in the laboratory. J Exp Mar Biol Ecol. 1984;79(2):129–47. 10.1016/0022-0981(84)90214-4

[9] Pratchett MS, Caballes CF, Wilmes JC, Matthews S, Mellin C, Sweatman HPA, et al Thirty years of research on Crown-of-Thorns starfish (1986–2016): scientific advances and emerging opportunities. Diversity. 2017;9(41). 10.3390/d9040041

[10] Babcock RC, Dambacher JM, Morello EB, Plagányi EE, Hayes KR, Sweatman HPA, et al Assessing different causes of Crown-of-Thorns Starfish outbreaks and appropriate responses for management on the Great Barrier Reef. PLoS ONE. 2016;11(12):e0169048 10.1371/journal.pone.0169048 28036360 PMC5201292

[11] Birkeland C. Terrestrial runoff as a cause of outbreaks of *Acanthaster planci* (Echinodermata, Asteroidea). Mar Biol. 1982;69(2):175–85. 10.1007/bf00396897

[12] Fabricius KE, Okaji K, De'ath G. Three lines of evidence to link outbreaks of the Crown-of-Thorns seastar *Acanthaster planci* to the release of larval food limitation. Coral Reefs. 2010;29(3):593–605. 10.1007/s00338-010-0628-z

[13] Brodie J, Devlin M, Lewis S. Potential enhanced survivorship of crown of thorns starfish larvae due to near-annual nutrient enrichment during secondary outbreaks on the central mid-shelf of the Great Barrier Reef, Australia. Diversity. 2017;9(17). 10.3390/d9010017

[14] Endean R, Stablum W. A study of some aspects of the Crown-of-Thorns starfish (*Acanthaster planci*) infestations of reefs of Australia's Great Barrier Reef. Atoll Res Bull. 1973;No. 167:1–20.

[15] Keesing JK, Halford AR, Hall KC. Mortality rates of small juvenile Crown of Thorns starfish *Acanthaster planci* on the Great Barrier Reef: implications for population size and larval settlement thresholds for outbreaks. Mar Ecol Prog Ser. 2018;597:179–90. 10.3354/meps12606

[16] Cowan Z-L, Pratchett M, Messmer V, Ling S. Known predators of Crown-of-Thorns starfish (*Acanthaster* spp.) and their role in mitigating, if not preventing, population outbreaks. Diversity. 2017;9(1):7 10.3390/d9010007

[17] Kroon FJ, Lefevre CD, Doyle JR, Patel F, Milton G, Severati A, et al DNA-based identification of predators of the corallivorous Crown-of-Thorns Starfish (Acanthaster cf. solaris) from fish faeces and gut contents. Sci Rep. 2020;10(1):8184 10.1038/s41598-020-65136-4 32424321 PMC7235266

[18] Sweatman H. No-take reserves protect coral reefs from predatory starfish. Curr Biol. 2008;18(14):R598–R9. 10.1016/j.cub.2008.05.033 18644332

[19] Vanhatalo J, Hosack GR, Sweatman H. Spatiotemporal modelling of crown-of-thorns starfish outbreaks on the Great Barrier Reef to inform control strategies. J Appl Ecol. 2017;54(1):188–97. 10.1111/1365-2664.12710

[20] Mellin C, MacNeil MA, Cheal AJ, Emslie MJ, Caley MJ. Marine protected areas increase resilience among coral reef communities. Ecol Lett. 2016;19(6):629–37. 10.1111/ele.12598 27038889

[21] Vine PJ. Crown-of-Thorns (*Acanthaster planci*) plagues: the natural causes theory. Atoll Res Bull. 1973;(166):1–10.

[22] Wolfe K, Graba-Landry A, Dworjanyn SA, Byrne M. Larval phenotypic plasticity in the boom-and-bust crown-of-thorns seastar, *Acanthaster planci*. Mar Ecol Prog Ser. 2015;539:179–89. 10.3354/meps11495

[23] Wolfe K, Graba-Landry A, Dworjanyn SA, Byrne M. Larval starvation to satiation: Influence of nutrient regime on the success of *Acanthaster planci*. PLoS ONE. 2015;10(3):e0122010 10.1371/journal.pone.0122010 25790074 PMC4366153

[24] Keesing JK, Halford AR. Importance of post-settlement processes for the population dynamics of *Acanthaster planci* (L.). Mar Freshwater Res. 1992;43(3):635–51. 10.1071/MF9920635

[25] Brodie JE. Enhancement of larval survival and recruitment in *Acanthaster planci* from the effects of terrestrial runoff: a review Aust J Mar Freshw Res. 1992;43(3):539–54. 10.1071/MF9920539

[26] Mellin C, Lurgi M, Matthews S, MacNeil MA, Caley MJ, Bax N, et al Forecasting marine invasions under climate change: Biotic interactions and demographic processes matter. Biol Conserv. 2016;204:459–67. 10.1016/j.biocon.2016.11.008

[27] Deaker DJ, Agüera A, Lin H-A, Lawson C, Budden C, Dworjanyn SA, et al The hidden army: corallivorous crown-of-thorns seastars can spend years as herbivorous juveniles. Biol Lett. 2020;16(4):20190849 10.1098/rsbl.2019.0849 32264781 PMC7211459

[28] Martinez AS, Byrne M, Coleman RA. Filling in the grazing puzzle: a synthesis of herboviry in starfish In: Hawkins SJ, Evans AJ, Dale AC, Firth, Hughes DJ, Smith IP, editors. Oceanogr Mar Biol. 55 Boca Raton: Crc Press-Taylor & Francis Group; 2017 p. 1–34. 10.1201/b21944-2

[29] Kamya PZ, Byrne M, Mos B, Dworjanyn SA. Enhanced performance of juvenile crown-of-thorns starfish in a warm-high CO_2_ ocean exacerbates poor growth and survival of their coral prey. Coral Reefs. 2018;37(3):751–62. 10.1007/s00338-018-1699-5

[30] Zann L, Brodie J, Berryman C, Naqasima M. Recruitment, ecology, growth and behavior of juvenile *Acanthaster planci* (L.) (Echinodermata, Asteroidea). Bull Mar Sci. 1987;41(2):561–75.

[31] Yamaguchi M. Coral reef asteroids of Guam. Biotropica. 1975;7(1):12–23. 10.2307/2989795

[32] Barker MF. Breeding and recruitment in a population of the New Zealand starfish *Stichaster australis* (Verrill). J Exp Mar Biol Ecol. 1979;41:195–211. 10.1016/0022-0981(79)90133-3

[33] Wilmes JC, Caballes CF, Cowan Z-L, Hoey AS, Lang BJ, Messmer V, et al Contributions of pre- versus post-settlement processes to fluctuating abundance of Crown-of-Thorns starfishes (*Acanthaster* spp.). Mar Pollut Bull. 2018;135:332–45. 10.1016/j.marpolbul.2018.07.006 30301045

[34] Johnson CR, Sutton DC, Olson RR, Giddins R. Settlement of crown-of-thorns starfish: role of bacteria on surfaces of coralline algae and a hypothesis for deep-water recruitment. Mar Ecol Prog Ser. 1991;71(2):143–62. 10.3354/meps071143

[35] Yamaguchi M. Early life histories of coral reef asteroids, with special reference to *Acanthaster planci* (L.) Biology and geology of coral reefs Vol 2 Biology 1 1–480: Academic Press, New York & London; 1973.

[36] Hoegh-Guldberg O. Uptake of dissolved organic matter by larval stage of crown-of-thorns starfish *Acanthaster planci*. Mar Biol. 1994;120(1):55–63. 10.1007/BF00381942

[37] Mellin C, Lugrin C, Okaji K, Francis DS, Uthicke S. Selective feeding and microalgal consumption rates by Crown-of-Thorns Seastar (*Acanthaster cf*. *solaris*) larvae. Diversity. 2017;9(1):8–Article No.:. 10.3390/d9010008

[38] Collins ARS. Biochemical investigation of two responses involved in feeding behaviour of *Acanthaster planci* (L.). III. food preferences. J Exp Mar Biol Ecol. 1975;17(1):87–94. 10.1016/0022-0981(75)90082-9

[39] Johansson CL, Francis DS, Uthicke S. Food preferences of juvenile corallivorous crown-of-thorns (*Acanthaster planci*) sea stars. Mar Biol. 2016;163(49). 10.1007/s00227-016-2823-0

[40] Keesing JK, Halford AR. Field measurement of survival rates of juveniles *Acanthaster planci*: Techniques and preliminary results. Mar Ecol Prog Ser. 1992;85(1):107–14. 10.3354/meps085107

[41] Lawrence JM, Lane JM. The utilization of nutrients by postmetamorphic echinoderms In: Jangoux M, Lawrence JM, editors. Echinoderm nutrition. Rotterdam, Netherlands: A.A. Balkema; 1982.

[42] Costerton JW, Lewandowski Z, Caldwell DE, Korber DR, Lappinscott HM. Microbial biofilms. Annu Rev Microbiol. 1995;49:711–45. 10.1146/annurev.mi.49.100195.003431 8561477

[43] Barker MF, Nichols D. Reproduction, recruitment and juvenile ecology of the starfish, *Asterias rubens* and *Marthasterias glasialis*. J Mar Biol Assoc UK. 1983;63(4):745–65. 10.1017/s0025315400071198

[44] Mead AD. On the correlation between growth and food supply in starfish. Am Nat. 1900;34:17–23. 10.1086/277530

[45] Haszprunar G, Vogler C, Wörheide G. Persistent gaps of knowledge for naming and distinguishing multiple species of crown-of-thorns-seastar in the *Acanthaster planci* species complex. Diversity. 2017;9(2). 10.3390/d9020022

[46] Vogler C, Benzie J, Lessios H, Barber PH, Worheide G. A threat to coral reefs multiplied? Four species of crown-of-thorns starfish. Biol Lett. 2008;4(6):696–9. 10.1098/rsbl.2008.0454 18832058 PMC2614177

[47] Lamare M, Pecorino D, Hardy N, Liddy M, Byrne M, Uthicke S. The thermal tolerance of Crown-of-Thorns (*Acanthaster planci*) embryos and bipinnaria larvae: implications for spatial and temporal variation in adult populations. Coral Reefs. 2014;33(1):207–19. 10.1007/s00338-013-1112-3

[48] R Core Team. R: a language and environment for statistical computing. Vienna, Austria: R Foundation for Statistical Computing; 2017.

[49] Pinheiro J, Bates D, DebRoy S, Sarkar D, R Core Team. {nlme}: Linear and Nonlinear Mixed Effects Models. 2019.

[50] Pohlert T. Calculate Pairwise Multiple Comparisons of Mean Rank Sums (PMCMR). R package 2014.

[51] Lenth RV. Least-Squares Means: The {R} Package {lsmeans}. Journal of Statistical Software. 2016;69(1):1–33.

[52] Wickham H, Chang WT, Henry L, Pedersen TL, Takahashi K, Wilke K, et al ggplot2: Create elegant data visualisations using the grammar of graphics. 3.0.0 ed. New York: Springer-Verlag; 2018.

[53] Wilmes JC, Schultz DJ, Hoey AS, Messmer V, Pratchett MS. Habitat associations of settlement-stage crown-of-thorns starfish on Australia's Great Barrier Reef. Coral Reefs. 2020:12 10.1007/s00338-020-01950-6

[54] Hereu B, Zabala M, Linares C, Sala E. The effects of predator abundance and habitat structural complexity on survival of juvenile sea urchins. Mar Biol. 2005;146(2):293–9. 10.1007/s00227-004-1439-y

[55] Yiu DS, Feehan CJ. Articulated coralline algae provide a spatial refuge to juvenile sea urchins from predatory crabs. Mar Biol. 2017;164(4):7 10.1007/s00227-017-3108-y

[56] Keesing JK, Halford AR, Hall KC, Cartwright CM. Large-scale laboratory culture of the crown-of-thorns starfish *Acanthaster planci* (L.) (Echinodermata: Asteroidea). Aquaculture. 1997;157(3–4):215–26. 10.1016/s0044-8486(97)00062-8

[57] Wilmes J, Matthews S, Schultz D, Messmer V, Hoey A, Pratchett M. Modelling growth of juvenile Crown-of-Thorns starfish on the northern Great Barrier Reef. Diversity. 2017;9(1). 10.3390/d9010001

[58] Kamya PZ, Byrne M, Graba-Landry A, Dworjanyn SA. Near-future ocean acidification enhances the feeding rate and development of the herbivorous juveniles of the Crown-of-Thorns starfish, *Acanthaster planci*. Coral Reefs. 2016;35(4):1241–51. 10.1007/s00338-016-1480-6

[59] Nagarkar S, Williams GA, Subramanian G, Saha SK. Cyanobacteria-dominated biofilms: a high quality food resource for intertidal grazers. Hydrobiologia. 2004;512(1–3):89–95. 10.1023/B:HYDR.0000020313.09924.c1

[60] Nichols D, Barker MF. Growth of juvenile *Asterias rubens* L. (Echinodermata, Asteroidea) on an intertidal reef in southwestern Britain. J Exp Mar Biol Ecol. 1984;78(1–2):157–65. 10.1016/0022-0981(84)90076-5

[61] Clements M, Wolfe K, Schwartz K, Byrne M. Forever fissiparous: asexual propagation and stable demography in a tropical and geographically isolated asterinid sea star. Mar Biol. 2019;166(6):11 10.1007/s00227-019-3518-030613111

[62] Allen JD, Richardson EL, Deaker D, Agüera A, Byrne M. Larval cloning in the crown-of-thorns sea star, a keystone coral predator. Mar Ecol Prog Ser. 2019;609:271–6. 10.3354/meps12843

[63] Messmer V, Pratchett M, Chong-Seng K. Variation in incidence and severity of injuries among Crown-of-Thorns Starfish (*Acanthaster cf*. *solaris*) on Australia's Great Barrier Reef. Diversity. 2017;9(12). 10.3390/d9010012

[64] Budden C, Butler I, Wolfe K, Deaker D, Sweatman H, Byrne M. Effect of sublethal predation on reproductive output of the crown-of-thorns starfish *Acanthaster* sp., with an overview of arm damage. Mar Ecol Prog Ser. 2019;629:103–16. 10.3354/meps13111

[65] Thorne BV, Byrne M. Survivorship of post-split fission products of *Holothuria atra* (Holothuroidea: Aspidochirotida) on the southern Great Barrier Reef. Invertebr Reprod Dev. 2013;57(4):293–300. 10.1080/07924259.2013.786762

[66] Lee J, Byrne M, Uthicke S. The influence of population density on fission and growth of *Holothuria atra* in natural mesocosms. J Exp Mar Biol Ecol. 2008;365(2):126–35. 10.1016/j.jembe.2008.08.003

[67] Byrne M. Changes in larval morphology in the evolution of benthic development by *Patiriella exigua* (Asteroidea, Asterinidae), a comparison with the larvae of *Patiriella* species with planktonic development. Biol Bull. 1995;188(3):293–305. 10.2307/1542306 29281334

[68] Soliman FES, Nojima S. Some observations on dispersal behaviour of the early juvenile stage of the sea-star, *Asterina minor* Hayashi. Publications from the Amakusa Marine Biological Laboratory Kyushu University. 1984;7(2):81–93.

[69] Nakamura M, Kumagai NH, Sakai K, Okaji K, Ogasawara K, Mitarai S. Spatial variability in recruitment of acroporid corals and predatory starfish along the Onna coast, Okinawa, Japan. Mar Ecol Prog Ser. 2015;540:1–12. 10.3354/meps11525

[70] Wilmes JC, Hoey AS, Messmer V, Pratchett MS. Incidence and severity of injuries among juvenile crown-of-thorns starfish on Australia’s Great Barrier Reef. Coral Reefs. 2019 10.1007/s00338-019-01830-8

[71] Birkeland C, Lucas JS. *Acanthaster planci*: major management problems of coral reefs. Boca Ranton, Florida: CRC Press Inc; 1990 1990.

[72] Brun E. Extreme population density of the starfish *Asterias rubens* L. on a bed of Iceland scallop, *Chlamys islandica* (O. F. Müller). Astarte. 1968;32:1–3.

[73] Witman JD, Genovese SJ, Bruno JF, McLaughlin JW, Pavlin BI. Massive prey recruitment and the control of rocky subtidal communities on large spatial scales. Ecol Monogr. 2003;73(3):441–62. 10.1890/01-4073

[74] Guillou M, Joly-Turquin G, Leyzour S, Pernet P, Dubois P. Factors controlling juvenile growth and population structure of the starfish *Asterias rubens* in intertidal habitats: field and experimental approaches. J Mar Biol Assoc UK. 2012;92(2):367–78. 10.1017/s0025315411001020

[75] Nauen CE. The growth of the sea star *Asterias rubens* and its role as benthic predator in Kiel Bay. Kieler Meers Sond. 1978:68–81.

[76] MacNeil MA, Chong-Seng KM, Pratchett DJ, Thompson CA, Messmer V, Pratchett MS. Age and growth of an outbreaking *Acanthaster cf*. *solaris* population within the Great Barrier Reef. Diversity. 2017;9(1):18 10.3390/d9010018

[77] Stump RJW, Lucas JS. Linear growth in spines from *Acanthaster planci* (L.) involving growth lines and periodic pigment bands. Coral Reefs. 1990;9(3):149–54. 10.1007/bf00258227

[78] Stump RJW. An investigation to describe the population dynamics of *Acanthaster planci* (L.) around Lizard Island, Cairns section, Great Barrier Reef Marine Park (CRC Reef Research Centre: James Cook University, Australia, 1996 CRC reef research technical report, Technical report no. 10.

[79] Wooldridge SA, Brodie JE. Environmental triggers for primary outbreaks of crown-of-thorns starfish on the Great Barrier Reef, Australia. Mar Pollut Bull. 2015;101(2):805–15. 10.1016/j.marpolbul.2015.08.049 26460182

[80] Hock K, Wolff NH, Condie SA, Anthony KRN, Mumby PJ. Connectivity networks reveal the risks of crown-of-thorns starfish outbreaks on the Great Barrier Reef. J Appl Ecol. 2014;51(5):1188–96. 10.1111/1365-2664.12320

[81] Reichelt RE, Greve W, Bradbury RH, Moran PJ. *Acanthaster planci* outbreak initiation: a starfish-coral site model Ecol Model. 1990;49:153–77. 10.1016/0304-3800(90)90026-d

[82] Gosselin LA, Qian PY. Juvenile mortality in benthic marine invertebrates. Mar Ecol Prog Ser. 1997;146(1–3):265–82. 10.3354/meps146265

[83] Lawrence JM, Moran P. Proximate composition and allocation of energy to body components in *Acanthaster planci* (Linneas)(Echinometra, Asteroidea) Zoological Science. 1992;9(2):321–8.

[84] Yamaguchi M. Population structure, spawning and growth of coral reef asteroid *Linckia laevigata* (Linneas). Pac Sci. 1977;31(1):13–30.

[85] Birkeland C. The faustian traits of the Crown-of-Thorns starfish. Am Scientist. 1989;77(2):154–63.

