# Supplementary material for: Diet flexibility and growth of the early herbivorous juvenile crown-of-thorns sea star, implications for its boom-bust population dynamics
Source: PLoS One. 2020 Jul 20;15(7):e0236142. doi: 10.1371/journal.pone.0236142 (PMC7371202; doi:10.1371/journal.pone.0236142)
Supplement: S1 Appendix — (DOCX) [file pone.0236142.s001.docx]

**S1 Appendix. Model of the exponential growth phase of herbivorous juvenile crown-of-thorns sea stars (COTS) on a diet of crustose coralline algae (CCA).**

The initial exponential growth phase (~ 1 – 10 mm Ø) of herbivorous juvenile COTS fed CCA was compared among sea stars in this study and two previous studies [1, 2]. Yamaguchi (1974) reared juveniles from 0.5 mm Ø to 11.0 mm Ø for 104 d [1]. Kamya et al. (2016) reared juveniles from 1.4 mm Ø to 4.2 mm Ø for 56 d [2]. The data modelled from this study included the cohorts of juveniles fed CCA for 104 d (1.7 – 10.3 mm Ø) and juveniles fed CCA for 133 d following 292 d on a diet of biofilm (3.3 – 10.5 mm Ø). An exponential growth equation was fit to each dataset using the lm function (stats package, formula: log(diameter) ~ time) in R ver. 3.4.3 [3]. To compare the rate of exponential growth among cohorts, the rate of growth (mm/day) was plotted against diameter (mm).

**References**

1. Yamaguchi M. Growth of juvenile *Acanthaster planci* (L.) in laboratory. Pac Sci. 1974;28(2):123-38.

2. Kamya PZ, Byrne M, Graba-Landry A, Dworjanyn SA. Near-future ocean acidification enhances the feeding rate and development of the herbivorous juveniles of the Crown-of-Thorns starfish, *Acanthaster planci*. Coral Reefs. 2016;35(4):1241-51. doi: 10.1007/s00338-016-1480-6.

3. R Core Team. R: a language and environment for statistical computing. Vienna, Austria: R Foundation for Statistical Computing; 2017.
