# Supplementary material for: Diet flexibility and growth of the early herbivorous juvenile crown-of-thorns sea star, implications for its boom-bust population dynamics
Source: PLoS One. 2020 Jul 20;15(7):e0236142. doi: 10.1371/journal.pone.0236142 (PMC7371202; doi:10.1371/journal.pone.0236142)
Supplement: S1 Table — Four cohorts of juveniles were raised on a diet of CCA in this study (CCA and biofilm, then CCA), Yamaguchi [1], and Kamya et al. [2] where D = diameter (mm) and t = time (days). The equation was differentiated (D’) to determine the equation for the rate of growth. DF, degrees of freedom. (DOCX) [file pone.0236142.s002.docx]

**S1 Table. Exponential models fitted to the growth of four cohorts of juvenile crown-of-thorns sea stars.** Four cohorts of juveniles were raised on a diet of CCA in this study (CCA and biofilm, then CCA), Yamaguchi [1], and Kamya et al. [2] where D = diameter (mm) and t = time (days). The equation was differentiated (D’) to determine the equation for the rate of growth. DF, degrees of freedom.

| Cohort | Coefficient | Intercept | DF | F-statistic | p-value | R^2^ | Growth equation | Rate of growth equation |
| --- | --- | --- | --- | --- | --- | --- | --- | --- |
| CCA | 0.018 | -2.337 | 1,3 | 875.9 | < 0.0001 | 0.995 | D = 0.097e^0.018t^ | D’ = 0.002e^0.018x^ |
| Biofilm, then CCA | 0.009 | -3.0833 | 1,6 | 293.3 | < 0.0001 | 0.977 | D = 0.046e^0.009t^ | D’ = 0.0004e^0.009x^ |
| Yamaguchi | 0.020 | -0.322 | 1,12 | 515.5 | < 0.0001 | 0.975 | D = 0.725e^0.020t^ | D’ = 0.015e^0.020x^ |
| Kamya et al. | 0.022 | -0.431 | 1,7 | 182 | < 0.0001 | 0.958 | D = 0.650e^0.022t^ | D’ = 0.014e^0.022x^ |
