# Supplementary material for: Diet flexibility and growth of the early herbivorous juvenile crown-of-thorns sea star, implications for its boom-bust population dynamics
Source: PLoS One. 2020 Jul 20;15(7):e0236142. doi: 10.1371/journal.pone.0236142 (PMC7371202; doi:10.1371/journal.pone.0236142)
Supplement: S2 Table — Four cohorts of juveniles were reared on CCA in this study (CCA, biofilm, then CCA), Yamaguchi [1], and Kamya et al. [2]. DF, degrees of freedom. (DOCX) [file pone.0236142.s003.docx]

**S2 Table. The linear relationship between the growth rate (mm/day) and the diameter juvenile crown-of-thorns sea stars.** Four cohorts of juveniles were reared on CCA in this study (CCA, biofilm, then CCA), Yamaguchi [1], and Kamya et al. [2]. DF, degrees of freedom.

| Cohort | Coefficient | Intercept | DF | F-statistic | p-value | R^2^ | Equation |
| --- | --- | --- | --- | --- | --- | --- | --- |
| CCA | 0.018 | -0.003 | 1,3 | 396.6 | 0.0003 | 0.990 | y = 0.02x - 0.003 |
| Biofilm, then CCA | 0.010 | -0.001 | 1,6 | 291.3 | < 0.0001 | 0.977 | y = 0.01x - 0.001 |
| Yamaguchi | 0.023 | -0.011 | 1,12 | 595.1 | < 0.0001 | 0.979 | y = 0.02x - 0.011 |
| Kamya et al. | 0.020 | 0.004 | 1,7 | 223.3 | < 0.0001 | 0.965 | y = 0.02x + 0.004 |
